# Frequency of anti-Chlamydia trachomatis antibodies in infertile women referred to Tabriz Al-Zahra hospital 

**Published:** 2017-01

**Authors:** Mahtab Sattari, Mehdi Ghiami Rad, Aaliye Ghasemzadeh, Zahra Mohammadoghli Reihan

**Affiliations:** 1 *Microbiology Department, Faculty of Basic Sciences, Islamic Azad University, Ahar Branch, Ahar, Iran.*; 2 *Department of Obstetrics and Gynecology, Faculty of Medicine, Tabriz University of Medical Sciences, Tabriz, Iran.*

**Keywords:** Chlamydia trachomatis, Infertile women, Tabriz

## Abstract

**Background:**

Infertility is one of the major issues in society and its incidence is estimated to be almost 10-15%. Chlamydia trachomatis (C. trachomatis) is an important cause of sexually transmitted diseases leading to infertility.

**Objective:**

This study was designed to determine the frequency of anti-C. trachomatis antibodies in infertile women at Al-zahra hospital, Tabriz, Iran.

**Materials and Methods:**

In this cross-sectional study, the blood samples were collected randomly from 184 infertile women (case group) and 100 pregnant women (control group). The frequency of specific IgG and IgM anti-C. trachomatis antibodies were evaluated using ELISA method.

**Results:**

The frequency of IgG anti-C. trachomatis antibody in the control and case groups was 18% and 35.88%, respectively. IgM anti-C. trachomatis antibody was found in 2% of controls and 5.44% of infertile women. Our results showed the significant differences between the case and control groups in anti-C. trachomatis antibodies (IgG, p=0.035 and IgM, p=0.004). Also, no significant relation was seen between the frequency of anti-C. trachomatis antibodies and age, location, and tubal factor infertility in our two study groups.

**Conclusion:**

According to high frequency of antibody anti-C. trachomatis among infertile women in competition to the control group, evaluation and treatment of Chlamydia infections is necessary in these patients.

## Introduction

Infertility is a social, economic and medical crisis that involved infertile couples live in all dimension (1). Infertility is the disability of being fertile after a regular intercourse for one year without prevention of pregnancy (2, 3). Infertility is one of the major issues in society and its incidence is estimated to be almost 10-15% (4). Infertility may have profound psychological effects. Many factors are involved in etiology of infertility. These factors are divided into three groups that include environmental, genetical, and infectious factors (5-7). Infectious factors can involve different parts of the genital system causing local or systemic effects that reduces the power of fertility (5, 8, 9). The incidence of infections in infertility is different from 39% in developed countries to 85% in African countries (10-12). 

*Chlamydia trachomatis* (*C. trachomatis*) genital infection is the most common sexually transmitted diseases in industrialized and developing countries (8, 9). It is also believed that chlamydial infections affect the outcome of infertility treatment and increase the risk of cervical cancer (11). According to World Health Organization (WHO) 90 million Chlamydial infections are found in the world annually (13). Chlamydias are intracellular bacteria living in epithelial cylindrical cells. Among Chlamydia species, *C. trachomatis* has the most effect on the reproduction system (14). *C. trachomatis* causes urethritis and cervicitis. Its complications include pelvic inflammatory disease, and infertility with tubal factor (14- 16). The tubal factor is one of the most important causes of infertility in women. The chlamydia symptoms are not chronic and may be hidden or undetectable under clinical condition. So when the patients became aware of their disease, the pathogen has left its complications (17). 

Many surveys were done about the role of *C. trachomatis* on infertility in women in Iran. In investigation by Badami *et al.*, a significant relation between *C. trachomatis* infection and women infertility was observed. In Nikbakht *et* *al.*, it was observed that anti *C. trachomatis* antibodies in infertile women with tubal f actor(25.27%) were significantly more than control group(12%) (p<0.05). (17, 18). 

Due to the importance of Chlamydia in infertility and genital infections and also investigating a complete survey about this in Tabriz, this study was aimed to determine the frequency of anti-Chlamydia ,antibodies in infertile women referring to Al-Zahra hospital, Tabriz, Iran.

## Materials and methods

In this cross-sectional study, blood samples were randomly collected (simple random sampling) from infertile women who were referred to Tabriz Al-Zahra hospital from November 2014 to April 2015. The sample size was calculated by Cochrane formula including 184 infertile women (case group) and 100 pregnant women (control group). The inclusion criteria were infertile women aged 16-40 yr. Women were examined by Gynecologist and patency of the fallopian tubes had been specified based on salpingography. The exclusion criteria were presence of chronic diseases like tuberculosis, and immunologic disorder, also antibiotic therapy during two weeks before sampling. Bloody serum samples were excluded. 

Information about location, age, duration of infertility, infertility in relatives and previous treatment or laboratory diagnosis proceeding was recorded filled by gynecologist. For determining IgG and IgM anti-*C. trachomatis* antibodies, 5 ml blood sample was taken by caped vacuum tube in a sterile condition. Samples were centrifuged for 10 min at 2000 rpm. The serum was transferred to a micro tube and kept in -7^o^C till performing the test. For determining of IgG and IgM anti-*C. trachomatis* antibodies in collected samples, ninety-six kit’s *C. trachomatis* ELISA IgG/IgM (Germany Vircell company) respectively with 98% sensitivity and 97% specialty for IgG and with 97% sensitivity and 97% specialty for IgM in ELISA and Elisa plate reader (Awareness 14; model 3200) were used. The results were compared with standard amount and density of antibodies and at last Optical Dencity (OD) was calculated by sample OD division OD cut off 10. Negative <9, 9-11 Equivocal positive ≥11.

**Ethical consideration**

The study protocol was approved by Tabriz University of Medical Science ethics committee. After explanation of study purpose and ensuring the confidentiality of their information, written infor consent was obtained from all participants. 

**Statistical analysis**

Statistical analysis was performed by Student’s *t*-test, Pearson test, One-way analysis of variance, and correlation coefficient in the level of significance P≤0.05 using SPSS software (Statistical Package for the Social Sciences, version 17, SPSS Inc, city, Illinois, country).

## Results

284 sera samples from case and control groups were examined using ELISA method. IgG anti-*C. trachomatis* antibody was positive in 18% of the control group and 35.88% of the case group (p=0.035). Also, 2% of the case group and 5.44% of controls were positive in IgM anti- *C. trachomatis* antibody (p=0.004). There was no significant relationship between the location and the prevalence of IgG and IGM anti- *C. trachomatis* antibodies (Table I and II). All participants were aged between 16-45 years and the samples were divided at four age levels (≤20, 21-31, 31-40, and >40 yr). There was no significant relationship between age and anti-*C. trachomatis* antibodies (IgG, p=0.437 and IgM, p=0.132). Also, no significant relation was seen between anti- *C. trachomatis* antibodies (IgG and IgM) and tubal factor infertility (Table III). 

**Table I:** Comparison of IgG and IgM anti- *C. trachomatis* antibodies titer in case (infertile women) and control (pregnant women**)** groups

**Antibody titer**	**IgG**		**IgM**	
	** Case group**	**Control group**	** Case group**	**Control group**
<9 (Negative)	100 (54.34)	68 (68)	170 (92.39)	94 (94)
9 to <11 (Equivocal)	18 (9.78)	14 (14)	4 (2.17)	4 (4)
11 (Positive)	66 (35.88)	18 (18)	10 (5.44)	2 (2)
Total	184 (100)	100 (100)	184 (100)	100 (100)
**p-value** *	0.004			0.035

All data presented as n (%).

* Independent Samples Test & Levene's Test for Equality of Variances

**Table II:** Comparison of IgG and IgM anti*- C. trachomatis* antibodies titer according to location in case (infertile women) and control (pregnant women

**Antibody titer**	**IgG**		**IgM**	
	**City**	**Village**	**City**	**Village**
<9 (Negative)	132	36	198	66
9 to <11 (Equivocal)	20	12	8	0
11 (Positive)	62	22	8	4
Total	214	70	214	70
p-value*	0.690		0.486	

SDs: 0.24806

* One-way ANOVA analyze test

**Table ІІI:** Tubal factor and anti-*c. trochomatis* antibodies in case and control group

**Pregnancy history**	**Fallopian tubes**			**Total**	**p-value** *	
	**Both opened**	**Both closed**	**1 opened/ 1 closed**		**IgG**	**IgM**
control group	100	0	0	100	0.208	0.082
case group	158	10	16	184		
Total	258	10	16	284		

SDs: 0.0001

* One-way ANOVA was used for statistical analysis

## Discussion

Infertility is increasingly becoming a significant health problem in many areas of the world (19). Infections may cause fertility disorders by different mechanisms (20). The *C. trachomotis* infection is the most common sexually transmitted bacterial infection worldwide, especially among young adults, since 1996, its population is annually increased 20% (19). This study aimed to compare the antibody IgG, IgM anti-*C. trachomatis* in infertile and pregnant women. 

In this study, anti-*C. trachomatis* IgG and IgM antibodies titer in infertile women in comparison with fertile women showed a significant difference (p<0.05). Our results were similar to Sonmez *et al*, Ome-Aghoyo Lo *et al*, Jahromi *et al* and Siemery *et al *(21-24). While the results were not in line with Rashidi *et al* (25). We found no significant relationship between age and anti-*C. trachomatis* antibodies but people with age range of 21-30 yr old and 31-40 yr old had more bacteria pollution than the other groups. From this point, the results are almost similar to Chen *et al* and Nikbakht *et al* (18, 26).

In the present study, no significant relationship was found between the location (rural or urban) of infertile women and intensity and amount of anti-*C. trachomatis* antibody. Our data showed that no significant relationship was observed between the tubal factor infertility and the amount of anti-C. *trachomatis* antibody, which was similar to Basirat *et al*, while this was contrary to Sharma *et al*, Keltz *et al*, Idahl *et al*, Surana *et al* studies (19, 27-30). The difference in the results of some previous studies may be due to differences in sample size, the use of various diagnostic methods, different sociocultural conditions or due to differences in the used kit. lack of patients follow-up after their medication, and also lack of data regarding the husbands of patients in order to measure their serums anti- *C. trachomatis* antibody level were the limitations of this study.

## Conclusion

According to the results of this study, there is relationship between chlamydial infection and infertility. It is recommended to diagnose genital *C. trachomatis* infection in infertile women
